# *Agathis robusta* Bark Essential Oil Effectiveness against COVID-19: Chemical Composition, In Silico and In Vitro Approaches

**DOI:** 10.3390/plants11050663

**Published:** 2022-02-28

**Authors:** Maged E. Mohamed, Nora Tawfeek, Samar S. Elbaramawi, Eman Fikry

**Affiliations:** 1 Department of Pharmaceutical Sciences, College of Clinical Pharmacy, King Faisal University, Al-Ahsa 31982, Saudi Arabia; 2 Department of Pharmacognosy, Faculty of Pharmacy, Zagazig University, Zagazig 44519, Egypt; noratawfeek@zu.edu.eg (N.T.); efhassan@zu.edu.eg (E.F.); 3 Department of Medicinal Chemistry, Faculty of Pharmacy, Zagazig University, Zagazig 44519, Egypt; sselbaramawy@pharmacy.zu.edu.eg

**Keywords:** antiviral, docking, protease, RNA-dependent RNA polymerase, SARS-CoV2, spike receptor-binding domain

## Abstract

Severe Acute Respiratory Syndrome Coronavirus 2 (SARS-CoV2), the causative agent of Coronavirus Disease 2019 (COVID-19), has seriously threatened global health. Alongside the approved vaccines, the discovery of prospective anti-COVID-19 drugs has been progressively targeted. Essential oils (EOs) provide a rich source of compounds with valuable antiviral activities that may contribute as effective agents against COVID-19. In this study, the EO of *Agathus robusta* bark was investigated for its chemical composition and its antiviral activity against SARS-CoV2. Overall, 26 constituents were identified by gas chromatography-mass spectrometry (GC-MS) analysis. *α*-Pinene, tricyclene, *α*-terpineol, limonene, *d*-camphene, *trans*-pinocarveol, *α*-phellandren-8-ol, L-*β*-pinene and borneol were the major components. In silico docking of these constituents against viral key enzymes, spike receptor-binding domain (RBD), main protease (Mpro) and RNA-dependent RNA polymerase (RdRp), using Molecular Operating Environment (MOE) software revealed good binding affinities of the components to the active site of the selected targets, especially, the RBD. In Vitro antiviral MTT and cytopathic effect inhibition assays demonstrated a promising anti SARS-CoV2 for *A. robusta* bark EO, with a significant selectivity index of 17.5. The results suggested using this EO or its individual components for the protection against or treatment of COVID-19.

## 1. Introduction

Severe acute respiratory syndrome coronavirus 2 (SARS-CoV2) is the latest emerged contagious respiratory pathogen associated with the global outbreak of atypical pneumonia pandemic (COVID-19). It was discovered for the first time in December 2019 in Wuhan (China), sequenced by January 2020 and was announced as a pandemic disease by the World Health Organization (WHO) on 11 March 2020 [1,2]. Once exposed to SARS-CoV2, clinical symptoms may appear including fever, headache, runny nose, cough, sore throat and trouble breathing. However, most of the patients with SARS-CoV-2 showed normal and mild symptoms, and the mortality rate is lower than in its relatives: SARS-CoV and MERS-CoV [3]. SARS-CoV2 has been recognized as a positive-sense enveloped, single-stranded RNA virus from the *Coronaviridae* family [4]. Morphologically, the virion is spherical, having a central nucleus and jutting surface proteins through which the virus is entrenched in the lipid membranes from the host cells. One of these surface proteins was identified as a spike [S] protein that gives a characteristic solar-crown-like appearance to the viruses, thus the name coronaviruses (CoVs) [4]. 

Four genera (*α*-, *β*-, *γ*- and *δ*-) comprise the CoV family, two of which (*α*- and *β*- genera) are known to infect mammals, including humans, and to originate from the bat species, *Rousettus leschenaultia* [4,5,6]. SARS-CoV-2, aside from the severe acute respiratory syndrome (SARS-CoV-1) and the Middle East respiratory syndrome coronavirus (MERS-CoV), belongs to *β*- CoV family [1,6]. Accordingly, it shares the structural characteristics of the other *β*-coronaviruses, where the viral RNA encodes four major structural proteins, three of which are membrane proteins, the spike glycoprotein (S), the matrix protein (M) and the envelope protein (E), and the fourth is the nucleocapsid protein (N) that surrounds the genomic RNA. Furthermore, the Viral RNA encodes 16 other non-structural proteins (NSPs), including RNA-dependent RNA polymerase (RdRp), 3-chymotrypsin-like protease (3CLpro, known as coronavirus main protease (Mpro), and papain-like protease (PLpro). These main proteins and key enzymes are attractive therapeutic targets for the inhibition of SARS-CoV-2 [7,8,9,10,11].

A potential target to be considered is the spike glycoprotein (S-protein) which mediates the access of SARS-CoV-2 into the host cell and consists of two operative subunits, S1 and S2. The S1 subunit, the spike’s head, accommodates the receptor-binding domain (RBD), which binds the virus surface S-protein with its receptor, the host angiotensin-converting enzyme 2 (ACE2). The S2 subunit develops the spike trunk and mediates the consequent fusion of the viral envelope and host cellular membranes [12,13,14,15,16]. Therefore, the binding between the S-protein and ACE2 can be inhibited by the blockage of the RBD, resulting in banning the SARS-CoV-2 fusion with the host cell. Accordingly, the S-protein has emerged as an important target against SARS-CoV-2 infection [2].

Once the virus gains entry to the host cells, the viral RNA is released and then translated into viral polyproteins, which are subsequently exposed to cleavage into effector proteins using the viral main protease (Mpro). On the other hand, RdRp produces a full-length negative-strand RNA template to be used to generate more viral genomic RNA [17,18]. Therefore, Mpro is considered as a key enzyme in the viral polyprotein proteolytic process, in addition to the viral genome replication and transcription [19]. Targeting Mpro leads to the inhibition of viral maturation and potentiates the host immune response [11]. Moreover, RdRp exhibits a vital role in viral transcription and replication. Consequently, these enzymes have been assigned as desirable targets to discover novel antiviral approaches [20].

In addition to vaccine development, approaches targeting the viral block are a major aim. Meanwhile, researchers are focused on natural products as prospective sources of substances with anti-SARS-CoV2 potential [21,22]. Essential oils (EOs) have demonstrated a complicated matrix of volatile components belonging to various chemical classes involving monoterpenes, sesquiterpenes and phenylpropanoids. EOs have been reported to possess antiviral activity against a broad range of viruses including human immunodeficiency virus (HIV), human herpesviruses (HSV), influenza virus (IFV), avian influenza and yellow fever virus [23]. The lipophilic structure of EOs permits them to invade the viral envelope, which, in consequence, modulates the fluidity of the viral lipid double layer membranes allowing the disturbance of the viral life cycle [24]. 

Antiviral activities of EOs have been speculated through many mechanisms, including immediate effects on free viruses, the blocking of different virus life cycle steps, i.e., fusion, replication and release from host cells, and the inhibition of key viral enzymes [23,25]. Owing to the distinct antiviral actions of EOs, they have been claimed as an efficient remedy against COVID-19. Therefore, several studies were established to assess the antiviral activities of EOs against SARS-CoV2 using various approaches such as In Vitro experiments, docking models or in clinical issues [26].

*Agathis robusta* (C. Moore ex F. Muell.) F. M. Bailey, commonly known as Kauri pine, Queensland Kauri or smooth-barked Kauri, is one of the most ancient coniferous trees belonging to family Araucariaceae and is native to the Bismarck Archipelago, New Guinea, and Queensland [27,28]. It is a monoecious, longstanding tree with straight trunk, attaining 50 m in height and 3 m in diameter, and has smooth brownish bark. [27,29,30]. Early in this century, Baker and Smith studied the steam volatile oils of Australian araucaroids, including *A. robusta* leaves, where *d*-*α*-pinene was noticed as the major constituent of the oil [31,32]. Again, the leaf EO of *A. robusta* growing in Australia was analyzed at the end of 20th century, and it consisted of mainly spathulenol (36.7%) and rimuene (5.6%) [33]. Recently, *A. robusta* growing in India was investigated for the chemical compositions of resin and leaf EOs, where 34 constituents were identified in the resin EO comprising isobornyl acetate (37.9%) and limonene (12.3%) as major constituents. On the other hand, the leaf oil composition was quite different, with a total of 43 identified constituents, including *β*-selinene (18.1%) and rimuene (14.2%) as major constituents [34]. 

The literature survey indicated that there is no attempt has been made until now to investigate the chemical composition and possible biological activities of the bark EO of *A. robusta*. Therefore, the present work aimed to explore of the chemical constituents of *A. robusta* bark EO growing in Egypt and to investigate the oil’s ability to inhibit SARS-CoV2 using in silico (on different virus key enzymes) and In Vitro approaches.

## 2. Results

### 2.1. Volatile Constituents in A. robusta Bark EO

**Figure 1:**
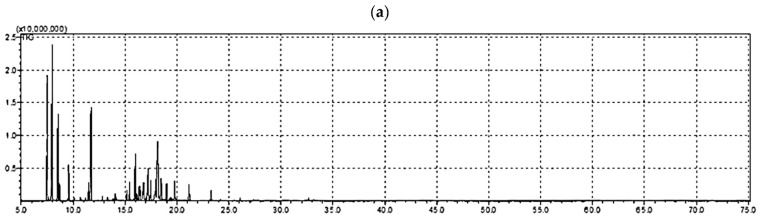

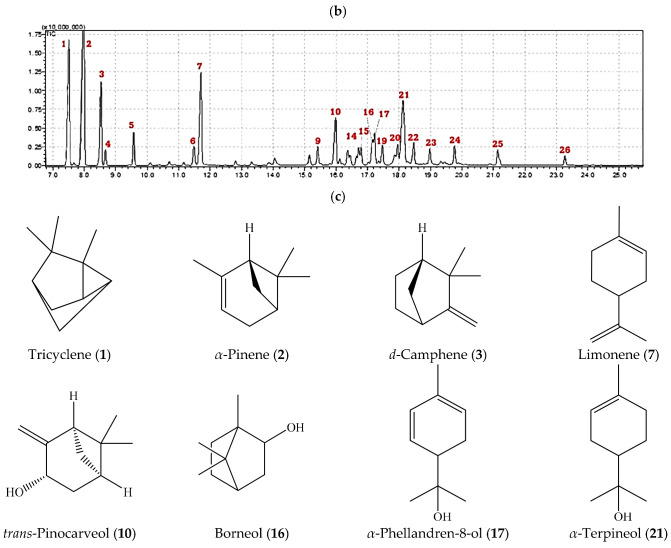
GC-MS chromatograms of the volatiles fraction extracted from *A. robusta* bark. (**a**) The whole GC-MS chromatogram. (**b**) The expanded GC-MS chromatograms from 7.0 to 25.3 min. (**c**) The structure of some GC-MS separated and identified major components of the *A. robusta* bark EO. Numbers in red (in (**b**,**c**)) are related to Table 1. The separation and identification conditions are explained in detail in the methods section.

The hydrodistilled EO obtained from *A. robusta* bark offered an average yield of 1.6 ± 0.07% v/dried weight (from three independent experiments). A total of 26 components, comprising 88.34% of the total composition, were identified and quantified using gas chromatography-mass spectrometry (GC-MS) analysis. The area percentages of identified compounds and their chemical names are illustrated in Table 1. The different separated essential oil structures as classified according to structure class, oxygenation and cyclization in Table 2. Figure 1 shows the GC/FID chromatogram of the *A. robusta* bark in a whole chromatogram view (Figure 1a) and a focused view (Figure 1b). The focused chromatogram view demonstrates the major identified compounds, and the structures of those compounds are revealed in Figure 1c.

### 2.2. Molecular Modelling and In Silico Study

#### 2.2.1. Molecular Docking Study

Three proteins of the COVID-19 virus, the main protease (Mpro), the RNA-dependent RNA polymerase (RdRp) and the virus spike receptor-binding domain (RBD), were implemented to provide insight on binding efficiencies of the *A. robusta* bark EO with the active sites of the targeted COVID-19 key targets. 

Re-docking the native ligands (N3: *N*-[(5-methylisoxazol-3-yl) carbonyl] alanyl-l-valyl-n-1-((1r,2z)-4-(benzyloxy)-4-oxo-1-{[(3r)-2-oxo-pyrrolidin-3-yl] methyl}c but-2-enyl)-l-leucinamide; peptide-like inhibitor) into the Mpro (PDB: 6LU7) protein revealed the validated active site, where RMSD is 1.9061 Å and energy score (S) is −8.4596 kcal·mol^−1^. Docking simulations of the major components of *A. robusta* bark EO inside the Mpro active site showed that all the compounds, tricyclene, *α*-pinene, *d*-camphene, limonene, *trans*-pinocarveol, borneol, *α*-phellandren-8-ol and *α*-terpineol, are deeply oriented in the pocket domain with reasonable values of root mean square deviation (RMSD) and docking score (S), Table S1 (Supplementary Materials). Moreover, *d*-camphene, limonene and *α*-phellandren-8-ol formed H–π bond interactions with His41. Hydroxyl groups of *trans*-pinocarveol, borneol and *α*-phellandren-8-ol exhibited hydrogen bond interactions with Met165, His164 and Met49, respectively.

Docking studies of tricyclene, *α*-pinene, *d*-camphene, limonene, *trans*-pinocarveol, borneol, *α*-phellandren-8-ol and *α*-terpineol on COVID-19 RNA-dependent RNA polymerase (RdRp) revealed that all the compounds reached the catalytic site of the enzyme. In comparison to the native ligand (H3U: 8-(3-(3-aminobenzamido)-4-methylbenzamido) naphthalene-1,3,5-trisulfonic acid), the docked components showed a fair binding affinity and lower RSMD resulting in more stable binding with lower rotations into the active site of RdRp. It was observed that the hydroxyl groups of *trans*-pinocarveol, borneol, *α*-phellandren-8-ol and *α*-terpineol formed hydrogen bond interactions with one of the key amino acid residues in the RdRp active site: Thr591, Table S2 (Supplementary Materials). 

**Figure 2:**
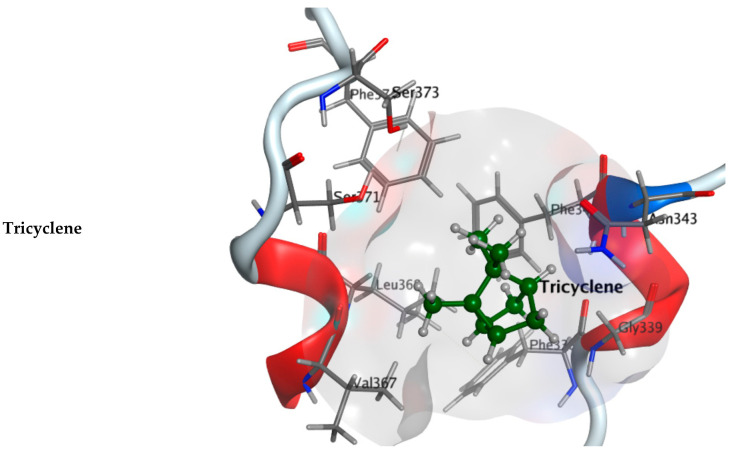

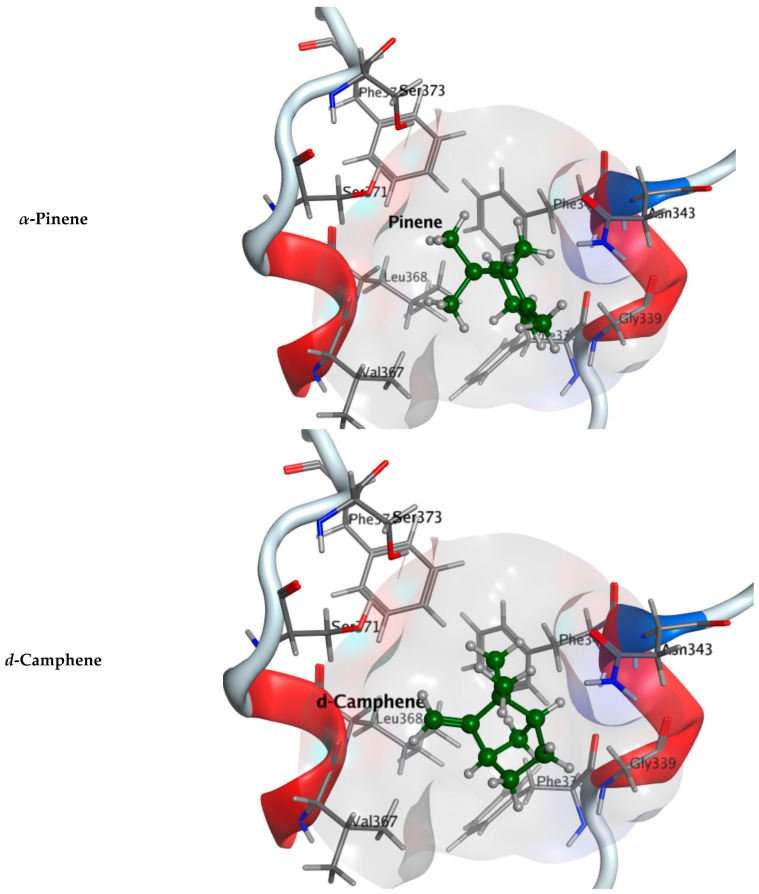

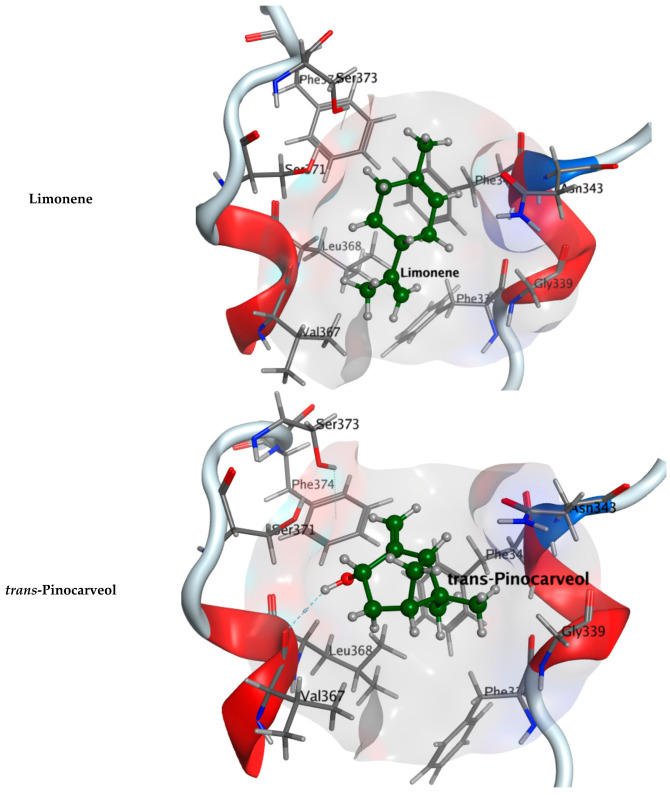

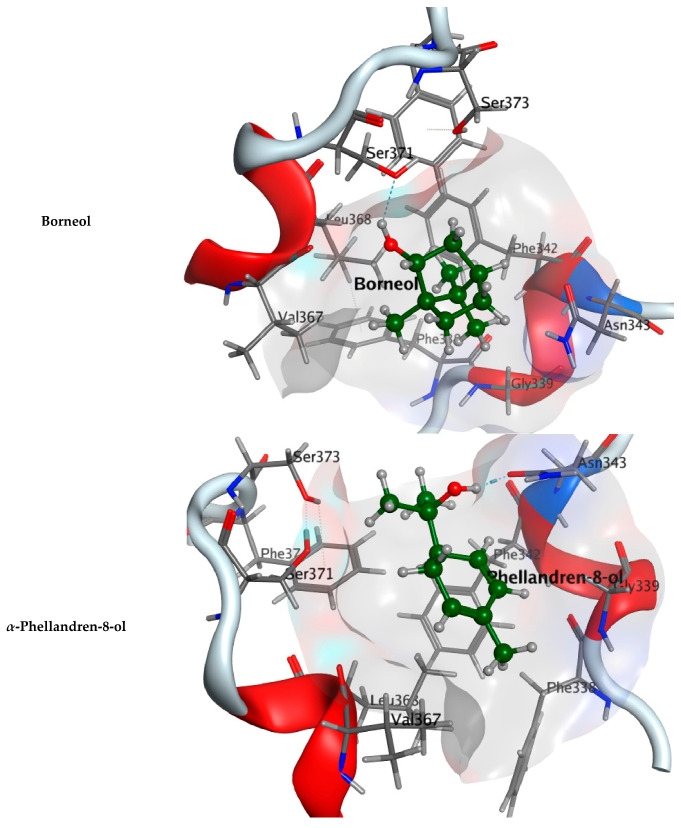

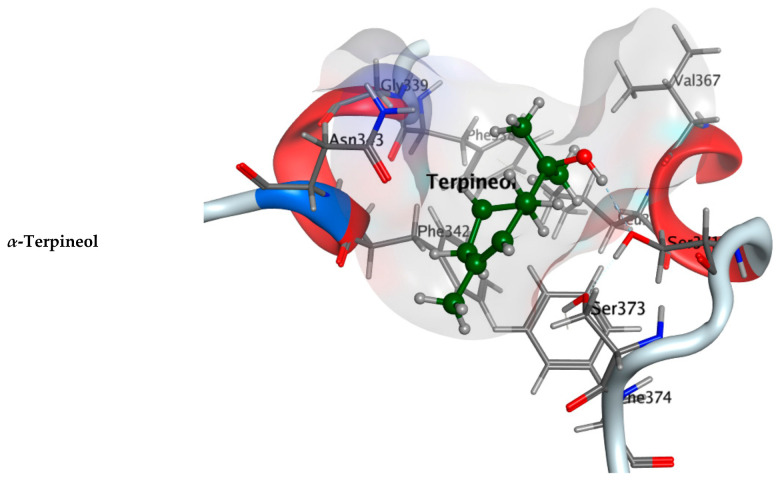
Three-dimensional binding mode of the major components of *A. robusta* bark EO inside the COVID-19 virus spike receptor-binding domain (PDB code: 7BZ5).

All 8 major components of *A. robusta* bark EO were docked into RBD (PDB: 7BZ5) with a good binding capacity and an affinity ranging from −3.8566 to −4.7246 kcal·mol^−1^ as the binding affinity of the co-crystallized ligand (NAG: 2-acetamido-2-deoxy-beta-D-glucopyranose) was −4.5304 kcal·mol^−1^. They fully occupied the active site of the receptor domain. Compared to the RBD co-crystallized ligand (RMSD: 1.5097), the RSMD of components of *A. robusta* bark EO range from 0.85503 to 2.1738, while the major components have RMSDs ranging from 1.1748 to 2.1738, Table S3 (Supplementary Materials). The hydroxyl group of *trans*-pinocarveol formed H-bond interactions with the Val367 of RBD, and hydroxyl groups of borneol, *α*-phellandren-8-ol and *α*-terpineol interacted with Asn343 through hydrogen bonds, Figure 2. Additionally, minor components also exhibited interactions with the key amino acid residues of the RBD active site, where camphor, *cis*-verbenol and myrtenal formed H-bonded interactions with Gly339, Ser371 and Leu 368, respectively. Another interesting finding is the H–π interactions between *m*-cymene, *trans*-pinocamphone, (-)-carvone and bornyl acetate and Phe338, Trp436, Phe342 and Trp436 amino acid residues, respectively, (Table S3 Supplementary Materials). 

The energy scores for binding of the major components with the three targeted COVID-19 enzymes are represented in Table 3.

#### 2.2.2. Flexible Alignments

The major components were examined for their conformational similarity with the co-crystallized ligand using the flexible alignment tool in Molecular Operating Environment (MOE). The best results of components are demonstrated in Table 4. As indicated from the 3D flexible alignment results, trans-pinocarveol, borneol, *α*-phellandren-8-ol and *α*-terpineol have similar conformations to the co-crystallized ligand.

The cyclic moiety in the components overlaps with the 2-deoxy-beta-D-glucopyranose moiety of the co-crystallized ligand. This elucidates that trans-pinocarveol, borneol, *α*-phellandren-8-ol and *α*-terpineol would share the same active site interactions. This was approved by the docking process as they exhibited hydrogen bond interactions with the key amino residues present in the active site.

### 2.3. ADME Assessment of the Major Components of A. robusta Bark EO

The absorption, metabolism and distribution parameters of tricyclene, *α*-pinene, *d*-camphene, limonene, *trans*-pinocarveol, borneol, *α*-phellandren-8-ol and *α*-terpineol were predicted using the SwissADME server. The calculated pharmacokinetic parameters are summarized in Table 5. Tricyclene, *α*-pinene, *d*-camphene and limonene have low gastrointestinal tract absorption. However, *trans*-pinocarveol, borneol, *α*-phellandren-8-ol and *α*-terpineol have high gastrointestinal absorption (GI) absorption. The prediction tool suggests the ability of compounds to cross the blood–brain barrier, indicating the increased absorption of compounds to CNS.

The computed metabolism of compounds showed that tricyclene, *α*-pinene, *d*-camphene, limonene, *trans*-pinocarveol, borneol, *α*-phellandren-8-ol and *α*-terpineol are not inhibitors for CYP1A2, CYP2C19, CYP2D6 and CYP3A4. The compounds, *α*-pinene, *d*-camphene and limonene were found as inhibitors of CYP2C9.

**Figure 3:**
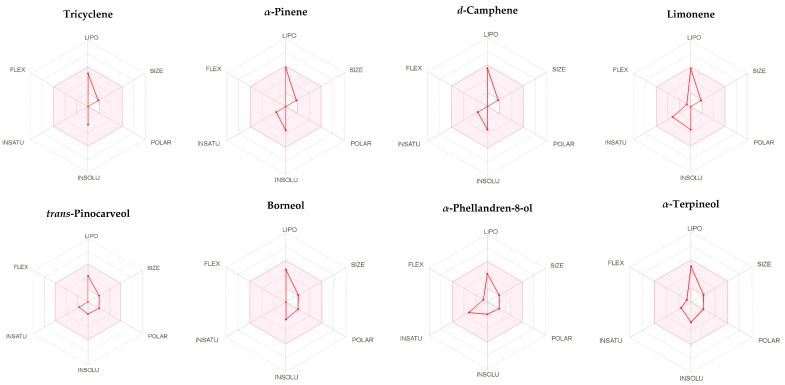
Bioavailability radar of the major components of *A. robusta* bark EO evaluated using the SwissADME web tool. The pink area represents the optimal range for each particular property for studied compounds (LIPO = lipophilicity as XLOGP3; SIZE = size as molecular weight; POLAR = polarity as TPSA (topological polar surface area); INSOLU = insolubility in water by log S scale; INSATU = insaturation as per fraction of carbons in the sp3 hybridization and FLEX = flexibility as per rotatable bonds).

Log *P*_o/w_ (MLOGP) for the compound lipophilicity should be lower than 4.15 so as not to violate the Lipinski’s rule. Limonene, *trans*-pinocarveol, borneol, *α*-phellandren-8-ol and *α*-terpineol do not violate Lipinski’s rule, as the MLOGP ranged from 2.20 to 3.27. However, tricyclene, *α*-pinene and *d*-camphene slightly violate the rule, as the MLOGP was > 4.15, ranging from 4.29 to 4.43. The pink region of the bioavailability radar (Figure 3) indicted a favorable properties range, Table 5. 

Overall, the components relatively fulfil the criteria of drug-likeness, suggesting that they are potential drug candidates.

### 2.4. Cytotoxicity and Antiviral Activity of A. robusta Bark EO

**Figure 4:**
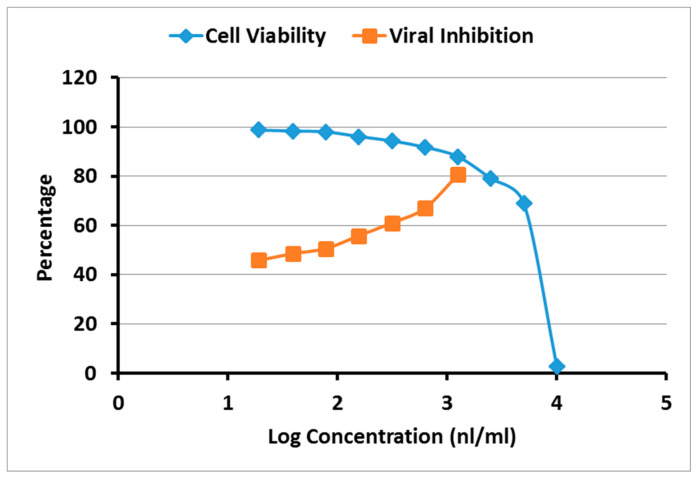
Dose-inhibition and dose-viability curves for *A. robusta* bark EO. The half maximal cytotoxic concentration (CC50) and 50% inhibitory concentration (IC50) were calculated through nonlinear regression analysis using GraphPad Prism software according to the materials and methods Section 4.6 and Section 4.7, respectively.

To evaluate the antiviral activity of *A. robusta* bark EO, the half maximal cytotoxic concentration (CC_50_) and the 50% inhibitory concentration (IC_50_) were calculated (Figure 4). The oil showed CC_50_ = 5.675 μL/mL and IC_50_ = 0.324 μL/mL. The overall therapeutic activity was determined by calculating the selectivity index (SI). The results revealed that *A. robusta* bark EO exhibited a promising In Vitro activity against NRC-03-nhCoV with a significant selectivity index (17.5) for antiviral activity relative to cellular toxicity.

## 3. Discussion

The current crisis of COVID-19 demonstrates the need for potential antiviral drug therapies. Scientists from several medical fields have a serious interest in the invention of new bio-active antiviral molecules from the EOs of aromatic medicinal plants as promising sources to prevent viral replication and infection.

*A. robusta* bark EO was analyzed for the first time by GC-MS. The composition of the bark EO disclosed that monoterpenes are the principal components of the oil, and it is almost devoid of sesquiterpenes constituents. *α*-pinene (19.49%), tricyclene (11.89%), *α*-terpineol (9.59%), limonene (9.37%), *d*-camphene (7.13%), *trans*-pinocarveol (4.95%), *α*-phellandren-8-ol (2.51%), L-*β*-pinene (2.36%) and borneol (2.32%) represent the major identified constituents of the oil. Oxygenation is observed among the oil components with 18 oxygenated compounds representing 35.16% of the total area percentage. Alcohols constitute the most common type of oxygenated components (9 compounds, 24.60%). Additionally, many cyclization patterns are detected, with most compounds being bicyclic (15 compounds, 46.75%) and monocyclic (10 compounds, 29.70%), with 1 tricyclic compound (11.89%) and none have been recognized as a tetracyclic or an acyclic compound (Table 1). Although the EO itself was not challenged before for its antiviral properties, some of its major components showed activities against many viruses, but not against the COVID-19 virus. *β*-pinene, limonene, *α*-terpinene, *γ*-terpinene, *α*-pinene, terpinen-4-ol, *α*-terpineol and 1,8-cineole revealed antiviral activity against the herpes simplex virus type 1 [36,37]. These EO components diminished the viral infectivity by more than 80% [37].

Several EOs and their isolated components have been described in In Vitro and in vivo models as effective antiviral agents against DNA and RNA viruses in different host cell lines by obstructing different steps of the viral life cycle [38]. These impacts are mainly based on the oil composition of mono- and sesquiterpene hydrocarbons together with phenolic, alcoholic, and other oxygenated other components [38,39]. Several EOs have been recognized for their antiviral activities against a diversity of viruses, including human immunodeficiency virus (HIV), human herpes virus (HSV), avian influenza A virus (H5N1), influenza A virus (H1N1) and Zika virus [23]. Additionally, modern studies disclosed that some EOs have exerted a huge antiviral activity against SARS-CoV2. For example, Eucalyptus oil and its active constituent (eucalyptol) exhibited a promising therapeutic potential in the prevention and treatment of COVID-19 [40]. Furthermore, garlic EOs and their isolated constituents, particularly diallyl sulphide and allyl trisulphide, lemon and geranium EOs and their derivative compounds exemplify potential natural antiviral agents, which hinder coronavirus entry into the human body [41]. Various plants have been proposed as potential sources for anti-SARS-CoV-2 EOs, including *Eugenia brasiliensis*, *Melissa officinalis*, *Cedrus libani*, *Zingiber zerumbet*, *Zataria multiflora* and *Vetiveria zizanoides* [42].

Isolated monoterpenes such as *α*-pinene and *α*-terpineol exerted significant antiviral activity against HSV-1, with elevated selectivity index, demonstrated by the inhibition of HSV-1 plaque formation [43]. *α*-Terpineol owns activity against IFV-A by delaying the early steps in the viral replication cycle [38]. *α*-Pinene and *β*-pinene inhibit the binding between RNA and the IBV N-protein, thereby possessing anti-IBV activity [44]. Moreover, in silico studies emphasized their activity against SARS-CoV-2 by binding to the active site of Mpro, human serine protease TMPRSS2 and spike (S) glycoprotein [38,43]. 

Limonene has potential antiviral activity against yellow fever, HSV-1, influenza and dengue virus by preventing viral replication, in addition to its activity against SARS-CoV-2 by suppressing ACE-2 receptors in HT-29 epithelial cells [38,45]. EOs which contain major components such as camphene and trans-pinocarveol display activity against HSV-1/HSV-2 and Coxsakie virus B3, respectively [46,47]. Borneol derivatives exhibit potent antiviral activity against influenza A virus and orthopoxvirus [48,49]. 

Virtual screening of these constituents was carried out using Molecular Operating Environment (MOE) software to explore their possibilities of creating effective docking with the spike receptor-binding domain (RBD), in addition to the viral vital enzymes, main protease (Mpro) and RNA-dependent RNA polymerase (RdRp). Moreover, 3D crystal structures of the possible ligands for these COVID-19 targets (PDB: 6LU7, 7D4F and 7BZ5) were utilized in molecular modelling design to elucidate the inhibitory activity of *A. robusta* bark EO against the virus. The molecular docking of major components of *A. robusta* bark essential oil with COVID-19 Mpro (PDB: 6LU7) demonstrated good binding affinity revealed by the docking scores ranging from −4.1916 to −5.0752 kcal/mol. Moreover, visual inspection of the docking poses showed that the major components could fit within the main cleft of Mpro active site through hydrogen bond formation with Met49, His164 and Met165 in addition to the H–pi bond interaction with His41. The docking results with COVID-19 RdRp show that major components of *A. robusta* bark essential oil bind to the active site of the targeted enzyme, so they could interfere with the viral RNA synthesis. Each component of *A. robusta* bark essential oil spike RBD show favorable binding affinities obtained from docking study in an energy score ranging of 3.8556 to −4.7246 kcal/mol in comparison with the native ligand (−4.5304 kcal/mol). More negative expected binding affinity suggested a stronger favorable protein–ligand complex. Binding of the components of *A. robusta* bark essential oil with the active pocket of the spike RBD of COVID-19 could interfere in the refolding of the COVID-19 spike, preventing COVID-19 spikes from binding with human ACE2 and inhibiting the process of viral infection. Moreover, ADME assessment revealed that the components relatively fulfill the criteria of drug-likeness, indicating the studied components of the oil as potential drug candidates. These results recommend using each component in this EO, especially the major ones or the whole EO in the treatment or protection of COVID-19. 

The in silico study encouraged investigation into the antiviral activity of the *A. robusta* bark EO, which was determined by calculating the half-maximal cytotoxic concentration (CC_50_) and the 50% inhibitory concentration (IC_50_) that required reducing the virus-induced cytopathic effect (CPE) by 50%. The overall therapeutic activity was determined in terms of the selectivity index (SI), which is the ratio of the CC_50_ to IC_50_. High SI values (>10) suggest the drug is a good candidate as antiviral drug and that the drug effect is more directed toward viral inhibition with less cytotoxic activity toward the host cell [50,51]. The results declared a promising In Vitro activity for *A. robusta* bark EO exhibited against SARS-CoV2 with a significant selectivity index (17.5) for antiviral activity relative to cellular toxicity. 

In a summary, *A. robusta* bark EO could be an auspicious drug candidate in treatment of COVID-19 infection. The EO could be used either as a prophylactic or on the inception of SARS-CoV2 infection to act as anti SARS-CoV2 through the interaction with the RBD resulting in the subsequent inhibition of the viral envelope fusion with host cellular membranes or through the inhibition of viral replication by targeting the viral Mpro and/or RdRp. Clinical trials in humans are necessary to assist realizing the role of *A. robusta* bark EO intake during COVID-19 infection.

## 4. Material and Methods

### 4.1. Plant Material

The fresh bark of the cultivated tree of *A. robusta* (50 years old in age) was collected at the cone maturation stage from El-Orman Botanical Garden, Giza, Egypt (July 2021). Eng. Therese Labib, Consultant of Plant Taxonomy at Ministry of Agriculture and the Former Director of Orman Botanical Garden, Giza, Egypt, kindly confirmed the identity of the plant. A voucher specimen (ZU-Ph-Cog-0100) was kept at the Herbarium of the Department of Pharmacognosy, Faculty of Pharmacy, Zagazig University. 

### 4.2. Isolation of Bark EO

The shade-dried bark of *A. robusta* (300 g) was pulverized into a course powder and then subjected to hydrodistillation by the Clevenger-type apparatus according to Perveen, et al. [52]. Hydrodistillation was performed under atmospheric pressure at about 100 °C for 4 h. The recovered volatile fraction was dried (anhydrous sodium sulphate), and the obtained EO samples were retained in brown vials (to protect photosensitive compounds) in the refrigerator (4 °C) until used.

### 4.3. GC-FID and GC-MS Analyses

The isolated bark EO was subjected to GC-MS analysis according to Perveen, et al. [52]. Gas chromatography/flame ionization (GC/FID) analysis was carried out using GC-2010 Plus (Shimadzu Corporation, Kyoto, Japan) gas chromatograph supplied with FID-2010 Plus detector and a split/splitless injector. The column was RTX-5MS^®^ fused silica capillary (0.25 μm film thickness and 30 m × 0.25 mm i.d.); the oven temperature was initially kept at 45 °C for 4 min and then increased to 270 °C at 4 °C/min and then held for 15 min. The carrier gas was helium with a flow rate of 2.0 mL/min; the temperatures of detector and injector were 300 and 250 °C, respectively, the split ratio was 1:20 and the injection volume is 1 μL. Quantification of EO components was accomplished by relative parentage area calculations. The relative percentage of the EO constituents was estimated on basis of the FID responses from the total peak area using percentage area normalization [53,54,55]. Gas chromatography/mass spectrometry (GC/MS) data were recorded on GCMS-QP2010 Plus (Shimadzu corporation, Kyoto, Japan). For the mass spectrometer, the ionization energy was 70 eV. The other conditions were identical to those mentioned for GC/FID; however, EI mass spectra were collected over the range of m/z 40–700 in full scan mode. Kovat’s retention indices (RI) were determined with respect to a set of co-injected standard hydrocarbons (C10–C28, Sigma Aldrich, Darmstadt, Germany). Identification of the compounds was carried out by comparing their spectral data and retention indices with Wiley Registry of Mass Spectral Data 10th edition (April 2013), NIST 11 Mass Spectral Library (NIST11/2011/EPA/NIH) and literature data [35]. The identified compounds and their percentages are listed in Table 1.

### 4.4. Molecular Modelling and In Silico Study

#### 4.4.1. Molecular Docking Study

Molecular docking of the major components of *A. robusta* bark EO (tricyclene, *α*-pinene, *d*-camphene, limonene, *trans*-pinocarveol, borneol, *α*-phellandren-8-ol and *α*-terpineol) was performed to provide insight on their binding efficiencies with the active sites of the selected SARS-COVID-19 enzymes. The molecular modelling studies of the components were carried out using Molecular Operating Environment MOE version 2019.0102 software (Chemical Computing Group, Montreal, QC, Canada) [56]. The docking placement methodology is triangle matcher, using London dG as the initial scoring function and GBVI/WSAdG as the final scoring function. The receptor was kept rigid, and the ligands were allowed to be flexible during the refinement process. The docking energy scores of the best-fitted poses of the ligands with the active site at the protein sites were recorded.

#### 4.4.2. Protein Preparation

Three proteins, COVID-19 main protease (Mpro) in complex with an inhibitor N3 (PDB: 6LU7), COVID-19 RNA-dependent RNA polymerase (RdRp) bound to suramin (PDB: 7D4F) and COVID-19 virus spike receptor-binding domain (RBD) complexed with a neutralized antibody (PDB: 7BZ5), were implemented to provide insight on binding efficiencies of the *A. robusta* bark EO with the active sites of the targeted COVID-19 enzymes. 

The crystal structures of the selected SARS-COVID-19 enzymes were retrieved from the RCSB Protein Data Bank website (http://www.rcsb.org (accessed on 28 October 2021)) [16] (PDB codes: 6LU7, 7D4F and 7BZ5) [18,57,58], Table S4. The crystalized water molecules were ignored from the enzyme complexes. Then, the protein structures were prepared individually using the MOE QuickPrep protocol for adding hydrogen atoms and partial charges through the Amber10: EHT forcefield.

#### 4.4.3. Ligand Preparation

The major components of *A. robusta* bark EO (tricyclene, *α*-pinene, *d*-camphene, limonene, *trans*-pinocarveol, borneol, *α*-phellandren-8-ol and *α*-terpineol) were drawn utilizing the Chemdraw^®^ program, then transferred to the MOE using smiles format. The energy of the components was minimized with root mean square (RMS) gradient 0.0001 kcal/mol and finally preparing a database file. The co-crystallized ligands were re-docked into the active site of the corresponding enzyme following the same set of the parameters as mentioned above for active site validation. Root mean square deviation (RMSD), docking energy score (S) and visual inspection of 2D and 3D planes of the component–targeted enzyme interactions were utilized for the analysis of the docking results.

#### 4.4.4. Flexible Alignments

Flexible alignment studies were performed on *trans*-pinocarveol, borneol, *α*-phellandren-8-ol and *α*-terpineol and the co-crystallized ligand of COVID-19 virus spike receptor-binding domain (RBD) using MOE 2019 software [56]. Flexible alignment mode and the resulting conformations were assessed according to the alignment score of the configuration (S). The latter is the sum of the similarity measure of configuration (F), and the average strain energy of the molecules in the alignment in kcal/mol (U). The lower S value indicates better alignment.

### 4.5. ADME Assessment of the Major Components of A. robusta Bark EO

The major components of *A. robusta* bark EO (tricyclene, *α*-pinene, *d*-camphene, limonene, *trans*-pinocarveol, borneol, *α*-phellandren-8-ol and *α*-terpineol) were subjected to ADME prediction using the SwissADME server using compounds in SMILES format. Swiss ADME [59], supported by SIB Swiss Institute of Bioinformatics, gives free access to several factors and predictive models to compute the physicochemical properties and estimate the pharmacokinetics and drug likeness of various small molecules.

### 4.6. MTT Cytotoxicity Assay

The half-maximal cytotoxic concentration (CC_50_) was assessed according to Mosmann [60]. The pure separated EO sample was serially diluted (*v/v*) to make 10 working solutions with Dulbecco’s Modified Eagle’s medium (DMEM). The 3-(4, 5-dimethylthiazol -2-yl)-2, 5-diphenyltetrazolium bromide (MTT) method, with minor modifications, was used to evaluate the cytotoxic activity of the *A. robusta* bark EO in VERO-E6 cells. Briefly, the cells were seeded in 96 well-plates (100 µL/well at a density of 3 × 10^5^ cells/mL) and then incubated for 24 h at 37 °C in 5% CO_2_. After 24 h, cells were treated with various concentrations of the tested sample in triplicates. After 24 h, the supernatant was discarded, cell monolayers were washed with sterile 1x phosphate buffer saline (PBS) 3 times and MTT solution (20 µL of 5 mg/mL stock solution) was added to each well and incubated at 37 °C for 4 h followed by medium aspiration. In each well, the formed formazan crystals were dissolved with 200 µL of acidified isopropanol (0.04 M HCl in absolute isopropanol = 0.073 mL HCL in 50 mL isopropanol). Absorbance of formazan solutions was measured at λ max 540 nm with 620 nm as a reference wavelength using a multi-well plate reader. The CC_50_ values were calculated using nonlinear regression analysis of GraphPad Prism software (version 5.01) by plotting log inhibitor versus normalized response (variable slope).

### 4.7. Inhibitory Concentration 50 (IC_50_) Determination

Determination of the inhibitory concentration 50 (IC_50_) was carried out as previously described by Mostafa, et al. [61]. In 96-well tissue culture plates, 2.4 × 10^4^ Vero-E6 cells were allocated in each well and incubated overnight at a humidified 37 °C incubator under 5% CO_2_ condition. The cell monolayers were then washed once with 1× PBS and exposed to virus adsorption (hCoV-19/Egypt/NRC-03/2020 (Accession Number on GSAID: EPI_ISL_430820)) for 1 h at room temperature (RT). The cell monolayers were further overlaid with 100 μL of DMEM containing different serial concentrations of *A. robusta* bark EO as specified above in working solution. After incubation at 37 °C in 5% CO_2_ incubator for 72 h, the cells were fixed with 100 μL of 4% paraformaldehyde for 20 min and stained with 0.1% crystal violet in distilled water for 15 min at RT. The crystal violet dye was then dissolved using 100 μL absolute methanol per well and the optical density of the color is measured at 570 nm using Anthos Zenyth 200rt plate reader (Anthos Labtec Instruments, Heerhugowaard, Netherlands). The IC_50_ of the sample is that required to reduce the virus-induced cytopathic effect (CPE) by 50%, relative to the virus control.

### 4.8. Calculation of Selectivity Index 

The selectivity index (SI) is a ratio the cytotoxic concentration of a drug to its effective bioactive concentration. The more elevated the SI ratio, the theoretically more efficient and safer a drug is. The SI value was calculated using the following equation:

SI = CC_50_/IC_50_.

## Figures and Tables

**Table 1:** Volatile constituents from *Agathis robusta* bark separated and identified after gas chromatography analysis.

No.	Compound Name ^a^	Rt ^b^	RI ^c^	Reported RI ^d^	Area Percentage
**1**	Tricyclene	7.509	927	921	11.89
**2**	*α*-Pinene	7.983	940	932	19.49
**3**	*d*-Camphene	8.529	953	946	7.13
**4**	2,4-Thujadiene	8.672	966	953	1.06
**5**	L-*β*-Pinene	9.57	983	974	2.36
**6**	*m*-Cymene	11.485	1030	1018	1.58
**7**	Limonene	11.707	1040	1029	9.37
**8**	*γ*-Terpinene	12.807	1068	1059	0.30
**9**	*α*-Campholenal	15.421	1140	1126	1.33
**10**	*trans*-Pinocarveol	15.988	1152	1135	4.95
**11**	Camphor	16.119	1162	1145	0.43
**12**	Camphene hydrate	16.447	1164	1146	0.73
**13**	*Trans*-Pinocamphone	16.642	1170	1158	0.66
**14**	Pinocarvone	16.72	1176	1160	1.40
**15**	Isoborneol	16.794	1177	1161	1.30
**16**	Borneol	17.156	1183	1165	2.32
**17**	*α*-Phellandren-8-ol	17.223	1186	1170	2.51
**18**	*cis*-Verbenol	17.351	1189	1172	0.29
**19**	L-terpinen-4-ol	17.471	1191	1174	1.66
**20**	Myrtenal	17.957	1204	1195	1.41
**21**	*α*-Terpineol	18.125	1208	1186	9.59
**22**	Verbenone	18.472	1214	1205	1.93
**23**	*trans*-Carveol	18.98	1229	1215	1.25
**24**	(-)-Carvone	19.767	1276	1239	1.39
**25**	Bornyl acetate	21.139	1304	1287	1.29
**26**	*α*-Terpineol acetate	23.272	1368	1346	0.72
**Total**					88.34

In elution order from RTX-5MS^®^ column. ^a^ Rt: retention time in minutes. ^b^ RI = identification based on retention index relative to standard *n*-alkanes. ^c^ All reported RI are from [35]. ^d^ The mean of three independent plant samples from the same area. Standard error of mean was removed to simplify reading and it did not exceed 10% of the mean value.

**Table 2:** *Agathis robusta* bark essential oil components classified according to the structures’ classes, the oxygenation and the cyclization. Serial numbers of structures mentioned in the second column are related to Table 1.

Oil Components Classified According to Common Structures	Serial Numbers of Compounds Belonging to Each Class	Area Percentage	Total Number of Compounds
Monoterpenes	1–26	88.37	26
Oxygenation			
Oxygenated	9–26	35.16	18
Alcohols	10, 12, 15, 16, 17, 18, 19, 21, 23	24.60	9
Aldehydes	9, 20	2.74	2
Esters	25, 26	2.01	2
Ketones	11, 13, 14, 22, 24	5.81	5
Cyclization			
Acyclic	-	-	-
Monocyclic	6, 7, 8, 9, 17, 19, 21, 23, 24, 26	29.7	10
Bicyclic	2, 3, 4, 5, 10, 11, 12, 13, 14, 15, 16, 18, 20, 22, 25	46.75	15
Tricyclic	1	11.89	1

**Table 3:** Energy score (s; kcal·mol^−1^) for binding of the major components with the three targeted COVID-19 enzymes.

Component Name	COVID-19 Mpro (PDB: 6LU7)	COVID-19 RdRp (PDB: 7D4F)	COVID-19 RBD (PDB: 7BZ5)
Tricyclene	−4.6674	−4.10620	−4.0962
*α*-Pinene	−4.7570	−4.2618	−3.8556
*d*-Camphene	−4.4746	−3.9284	−4.0584
Limonene	−4.7005	−4.5654	−3.9563
*trans*-Pinocarveol	−4.59218	−4.2639	−4.1561
Borneol	−4.1916	−4.0815	−3.9368
*α*-Phellandren-8-ol	−4.9801	−4.3839	−4.0385
*α*-Terpineol	−5.0752	−4.7213	−4.2190
Co-crystallized ligand	−8.4596	−7.7170	−4.5304

**Table 4:** Flexible alignment of co-crystallized ligand (NAG: 2-acetamido-2-deoxy-beta-D-glucopyranose in red color) with the following components (in green color).

Component	Flexible Alignment	S kcal/mol
*trans*-Pinocarveol	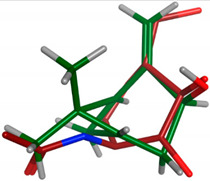	−146.505341
Borneol	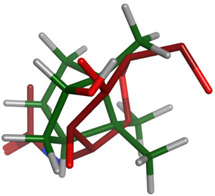	−22.2655106
*α*-Phellandren-8-ol	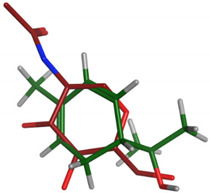	−48.4288139
*α*-Terpineol	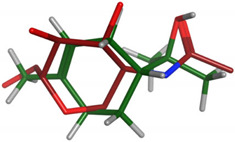	−51.053772

**Table 5:** Pharmacokinetics of the major components of *A. robusta* bark EO. The data were calculated in silico using the SwissADME web tool.

	Tricyclene	*α*-Pinene	*d*-Camphene	Limonene	*Trans*-Pinocarveol	Borneol	*α*-Phellandren-8-ol	*α*-Terpineol
M.wt.	136.23	136.23	136.23	136.23	152.23	154.25	152.23	154.25
HBA	0	0	0	0	1	1	1	1
HBD	0	0	0	0	1	1	1	1
GI absorption	Low	Low	Low	Low	High	High	High	High
BBB permeant	Yes	Yes	Yes	Yes	Yes	Yes	Yes	Yes
P-gp substrate	No	No	No	No	No	No	No	No
CYP1A2 inhibitor	No	No	No	No	No	No	No	No
CYP2C19 inhibitor	No	No	No	No	No	No	No	No
CYP2C9 inhibitor	No	Yes	Yes	Yes	No	No	No	No
CYP2D6 inhibitor	No	No	No	No	No	No	No	No
CYP3A4 inhibitor	No	No	No	No	No	No	No	No
Lipinski’s violation	1	1	1	0	0	0	0	0
MLOGP	4.43	4.29	4.29	3.27	2.30	2.45	2.20	2.30
Xlogp3	3.24	4.48	4.22	4.57	1.79	2.72	1.81	3.39
TPSA	0	0	0	0	20.23	20.23	20.23	20.23
Log S (ESOL), water solubility	−2.73	−3.51	−3.34	−3.50	−1.91	−2.51	−1.86	−2.87
Fraction Csp3	1	0.8	0.8	0.6	0.8	1	0.6	0.8
Num. rotatable bonds	0	0	0	1	0	0	1	1

HBA: num. H-bond acceptors, Num. H-bond donors: HBD, LIPO is Lipiphility: −0.7 < Xlogp3 < +5.0), SIZE (150 g/mol < M.wt. < 500 g/mol), POLAR (polarity): 20Å^2^ < TPSA < 130 Å^2^, INSOLU (insolubility): 0 < Log S (ESOL) < 6, INSAT (insaturation): 0.25 < fraction Csp3 < 1 and FLEX (Flexibility): 0 < Num. rotatable bonds < 9.

## Data Availability

Not applicable.

## Supplementary Materials

The following supporting information can be downloaded at: https://www.mdpi.com/article/10.3390/plants11050663/s1, Table S1: 3D-Binding mode of the major components of *A. robusta* bark EO inside the Mpro active site (PDB code: 6LU7); Table S2: 3D-Binding mode of the major components of *A. robusta* bark EO inside COVID-19 RNA-dependent RNA polymerase (PDB code: 7D4F); Table S3: Molecular docking details of *A. robusta* bark EO with RBD, Table S4: The target enzymes involved for docking of the major components of *A. robusta* bark EO.
