# Supplementary material for: Agathis robusta Bark Essential Oil Effectiveness against COVID-19: Chemical Composition, In Silico and In Vitro Approaches
Source: Plants (Basel). 2022 Feb 28;11(5):663. doi: 10.3390/plants11050663 (PMC8912836; doi:10.3390/plants11050663)
Supplement: Supplementary file 1 [file plants-11-00663-s001.zip › plants-1604000-supplementary.pdf]

Chemical composition of *Agathis robusta* bark essential oil: In silico and in vitro antiviral activities against COVID-19.

Maged E. Mohamed [1,2,*], Nora Tawfeek [2], Samar S. Elbaramawi [3], Eman Fikry [2]

[1] Department of Pharmaceutical Sciences, College of Clinical Pharmacy, King Faisal University, 31982 Al-Ahsa, Saudi Arabia; memohamed@kfu.edu.sa

[2] Department of Pharmacognosy, Faculty of Pharmacy, Zagazig University, Zagazig 44519, Egypt; noratawfeek@zu.edu.eg (N.T.); efhassan@zu.edu.eg (E.F.)

[3] Department of Medicinal Chemistry, Faculty of Pharmacy, Zagazig University, Zagazig 44519, Egypt; sselbaramawy@pharmacy.zu.edu.eg

* Correspondence: memohamed@kfu.edu.sa; Tel.: (optional; include country code; if there are multiple corresponding authors, add author initials)

Supplementary material

Table S1. 3D-Binding mode of the major components of *A. robusta* bark EO inside the Mpro active site (PDB code: 6LU7).

Component name	3D Protein- Ligand interaction	Energy score (S) (kcal/mol)/RMSD (Å)
Tricyclene	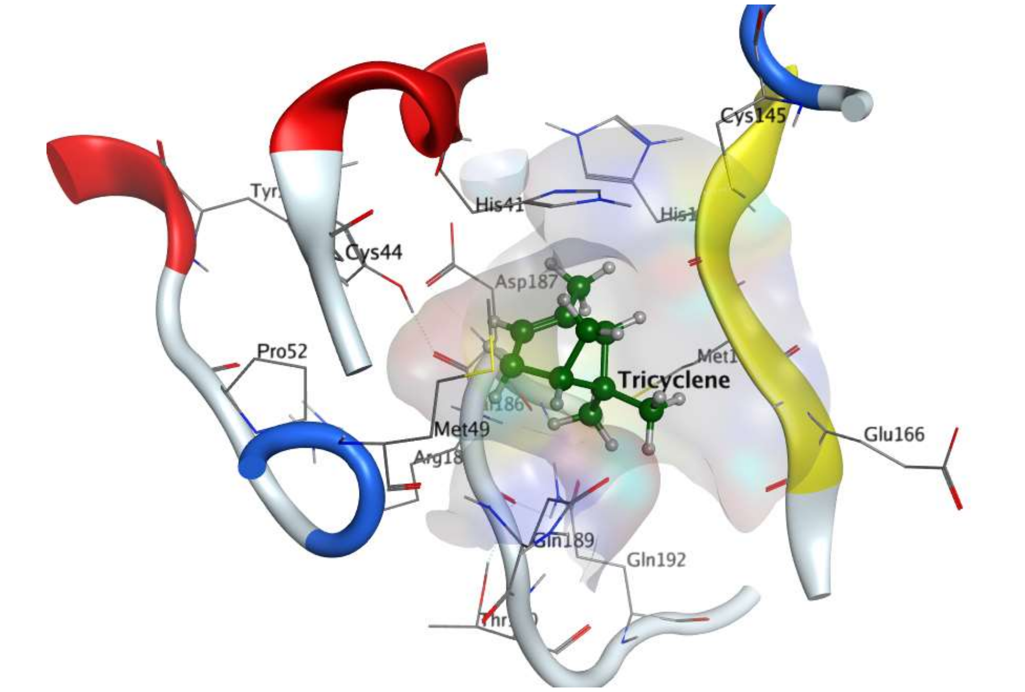	**-4.6674** **1.4627**
α-Pinene	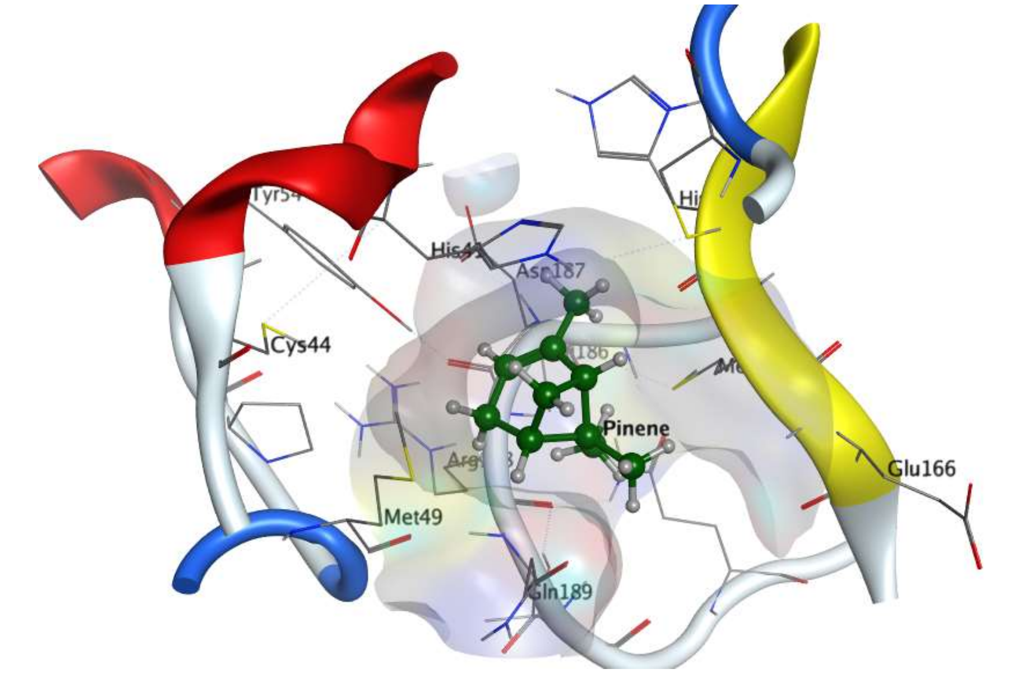	**-4.7570** **1.2320**

d-Camphene	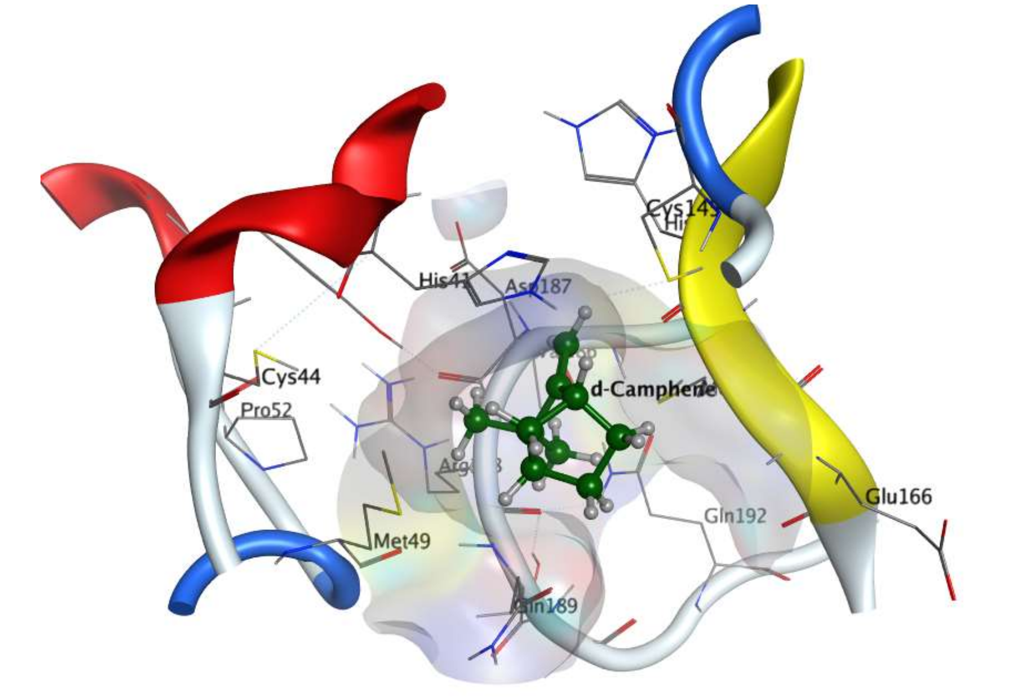	**-4.4746**<br><br>**1.7971**
**Limonene**	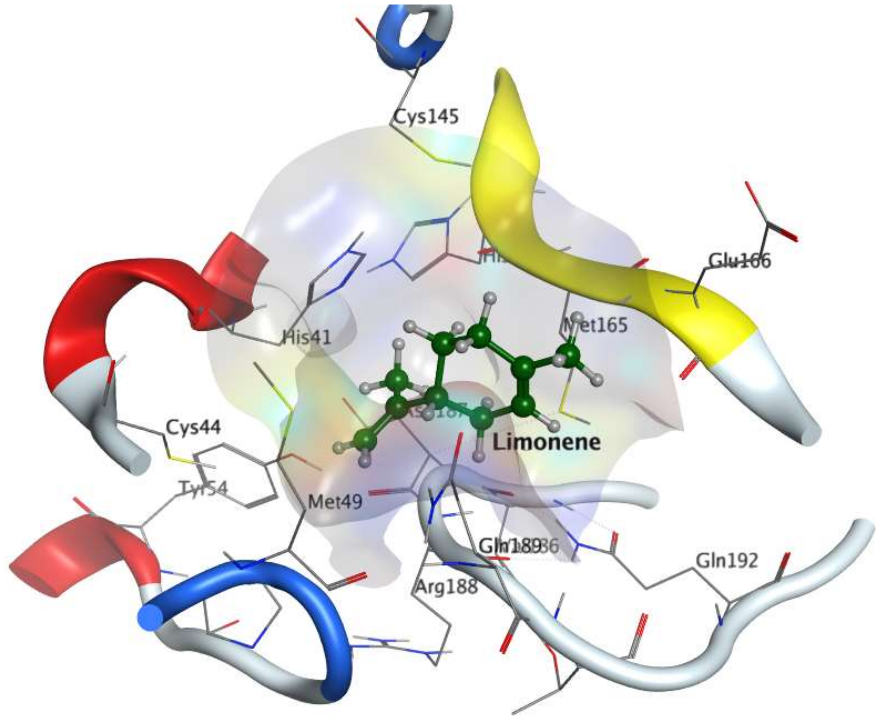	**-4.7005**<br><br>**1.6112**

<table>
<tr><td>trans-
Pinocarveol</td><td>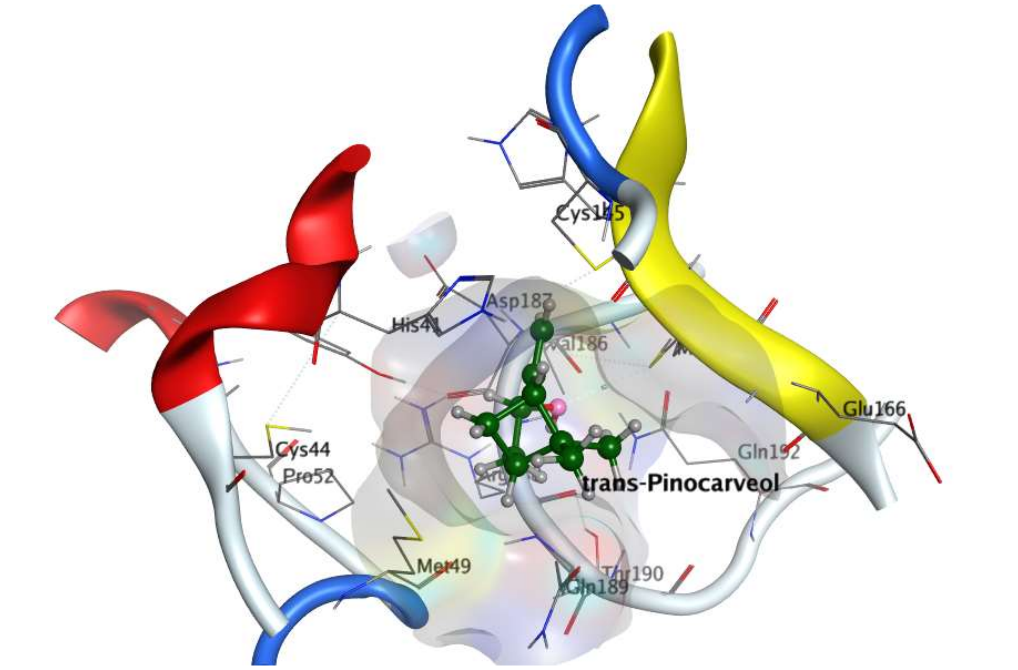
</td><td>-4.59218

2.0991</td></tr>
<tr><td>Borneol</td><td>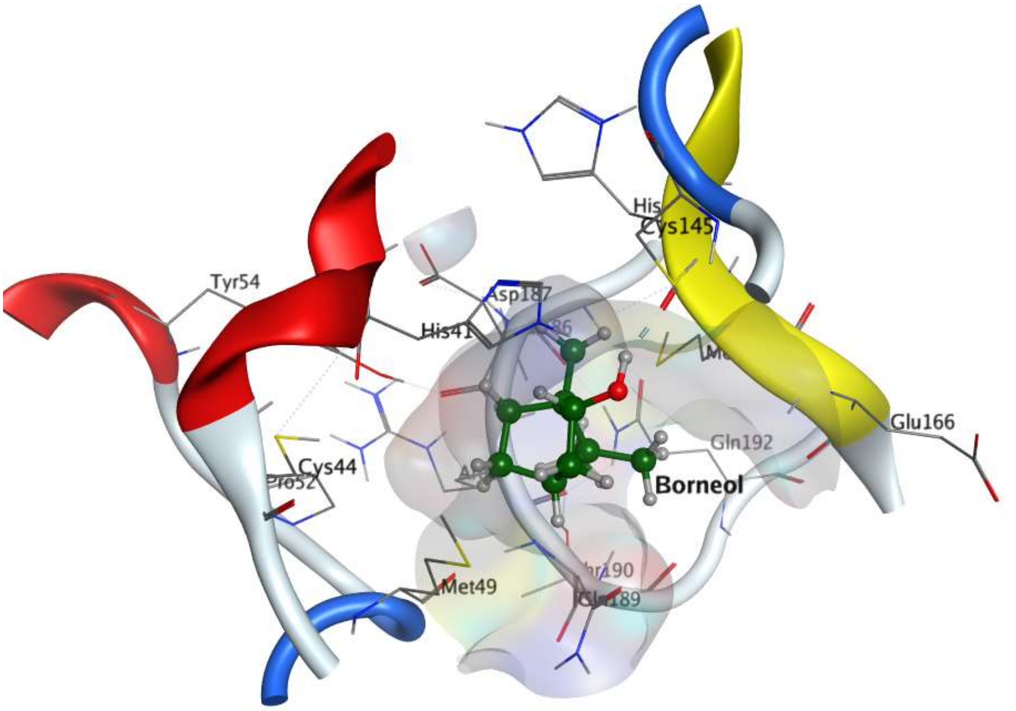
</td><td>-4.1916

1.1248</td></tr>
</table>

α-Phellandren-8-ol	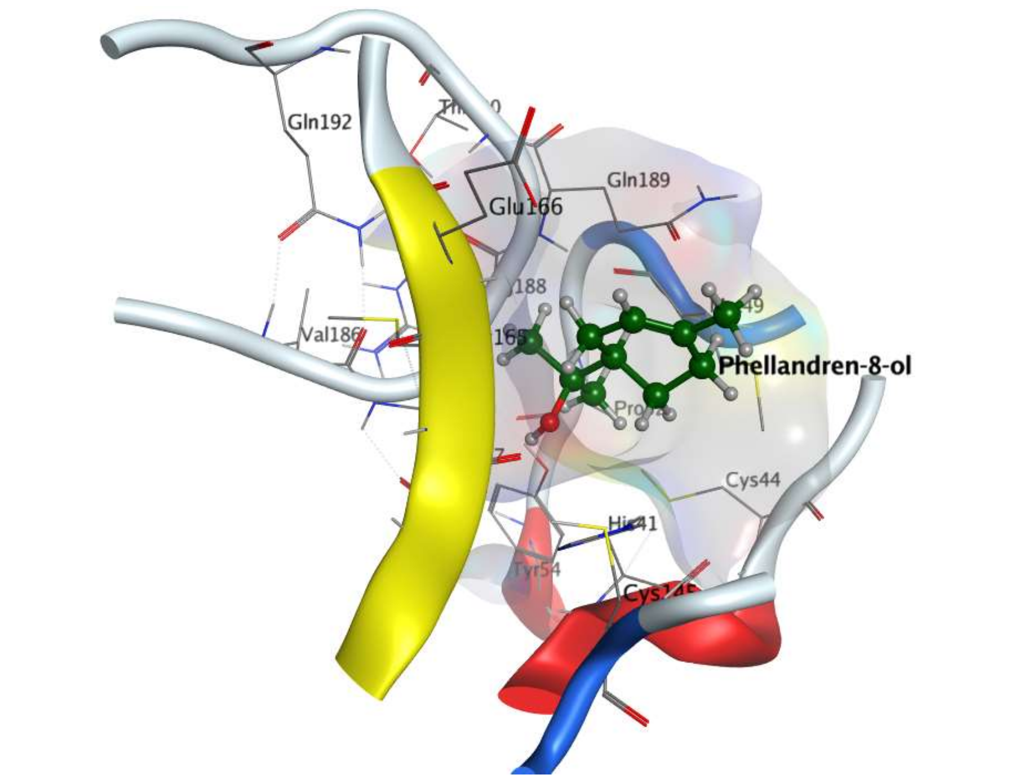	-4.9801<br><br>1.1112
α-Terpineol	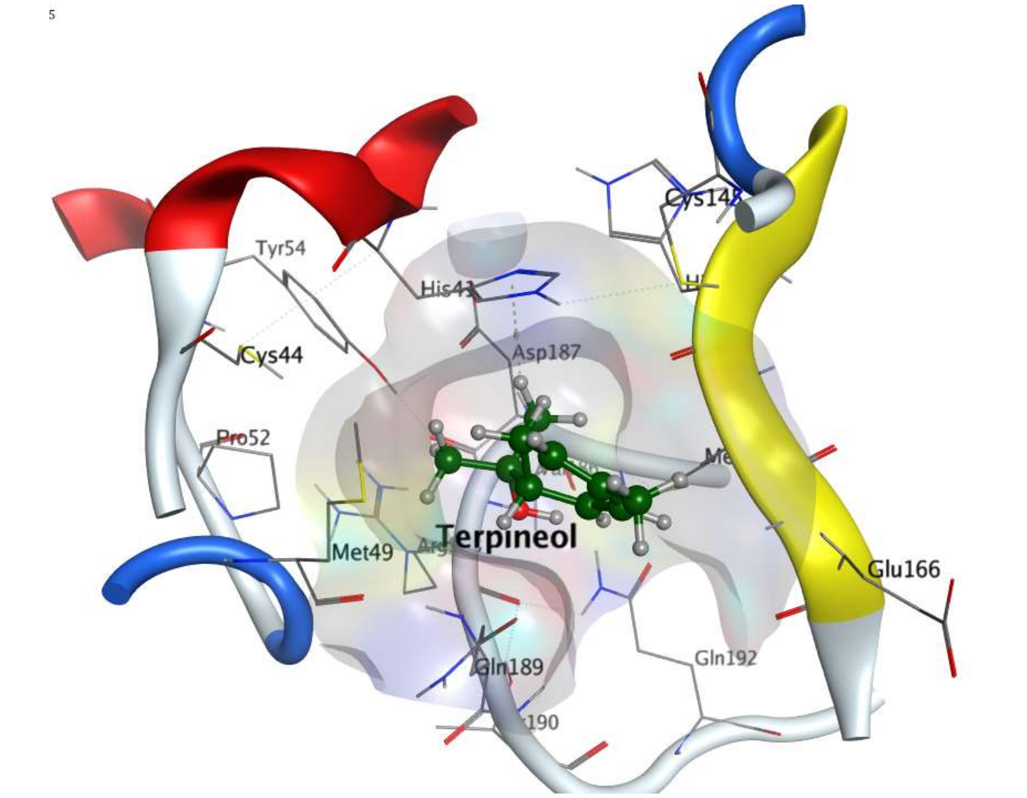	-5.0752<br><br>1.7314

Table S2. 3D-Binding mode of the major components of *A. robusta* bark EO inside Covid-19 RNA-dependent RNA polymerase (PDB code: 7D4F).

Component name	3D Protein- Ligand interaction	Energy score (S) (kcal/mol) /RMSD (Å)
**Tricyclene**	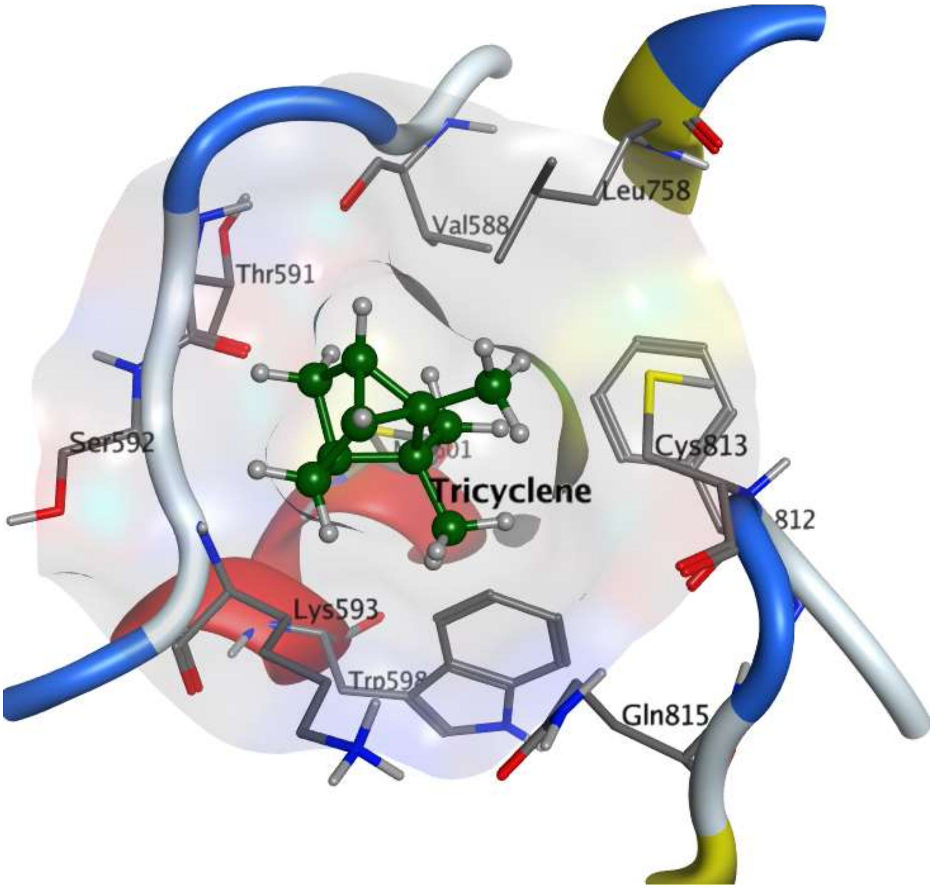	**-4.10620**<br><br>**2.0938**

α-Pinene	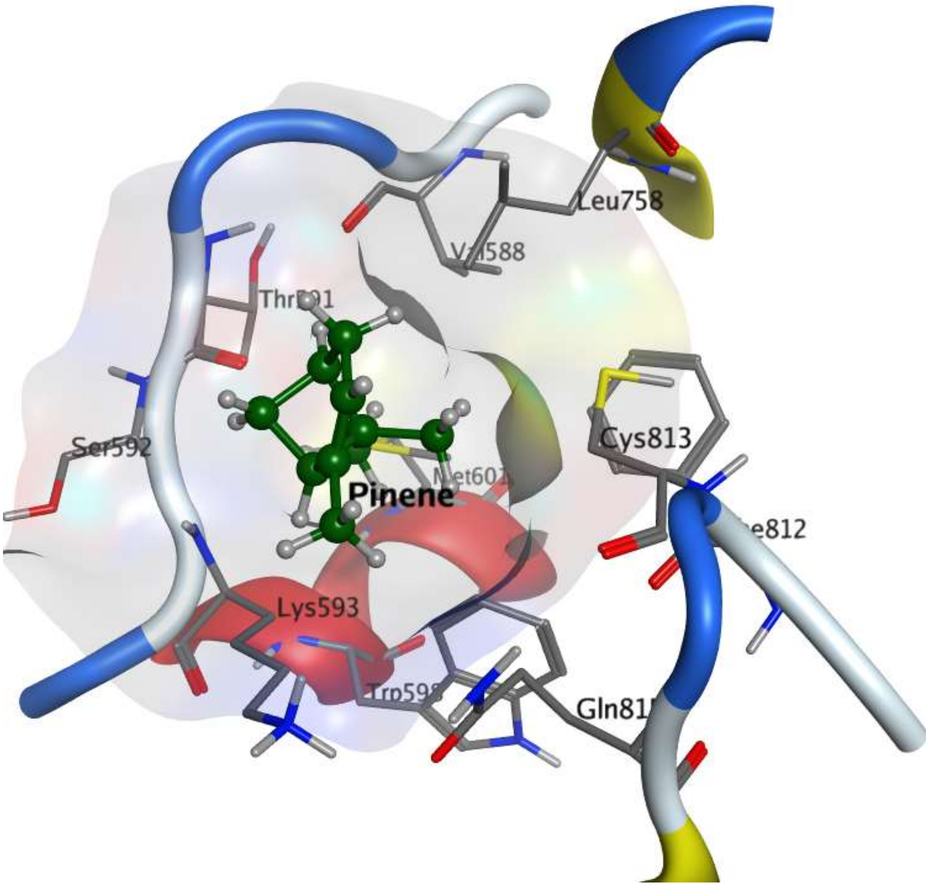	-4.2618<br><br>1.7163
d-Camphene	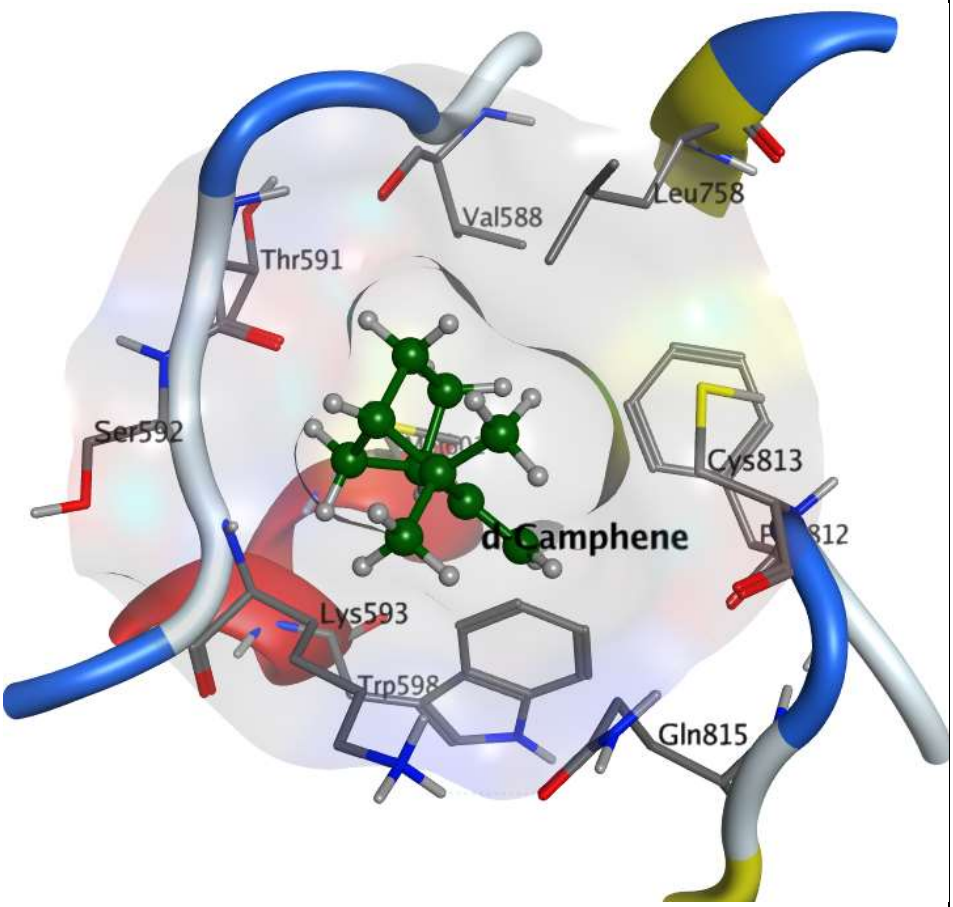	-3.9284<br><br>1.2590

**Limonene**	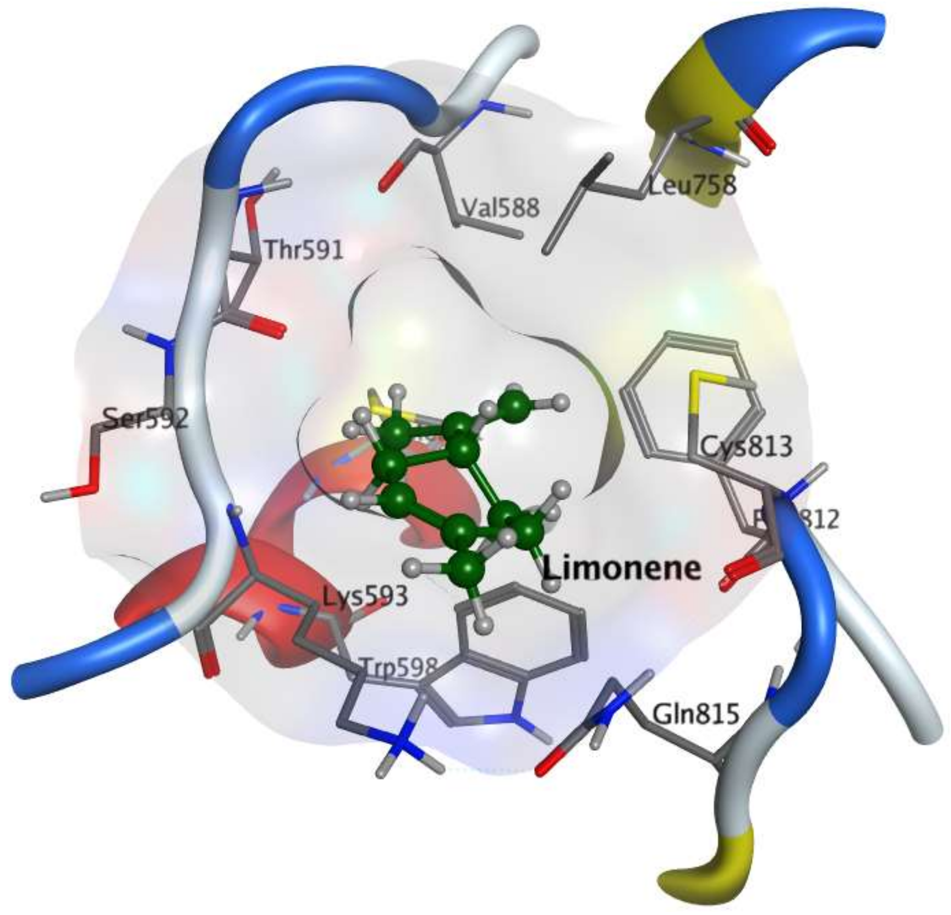	**-4.5654**<br><br>**0.9815**
***trans*-Pinocarveol**	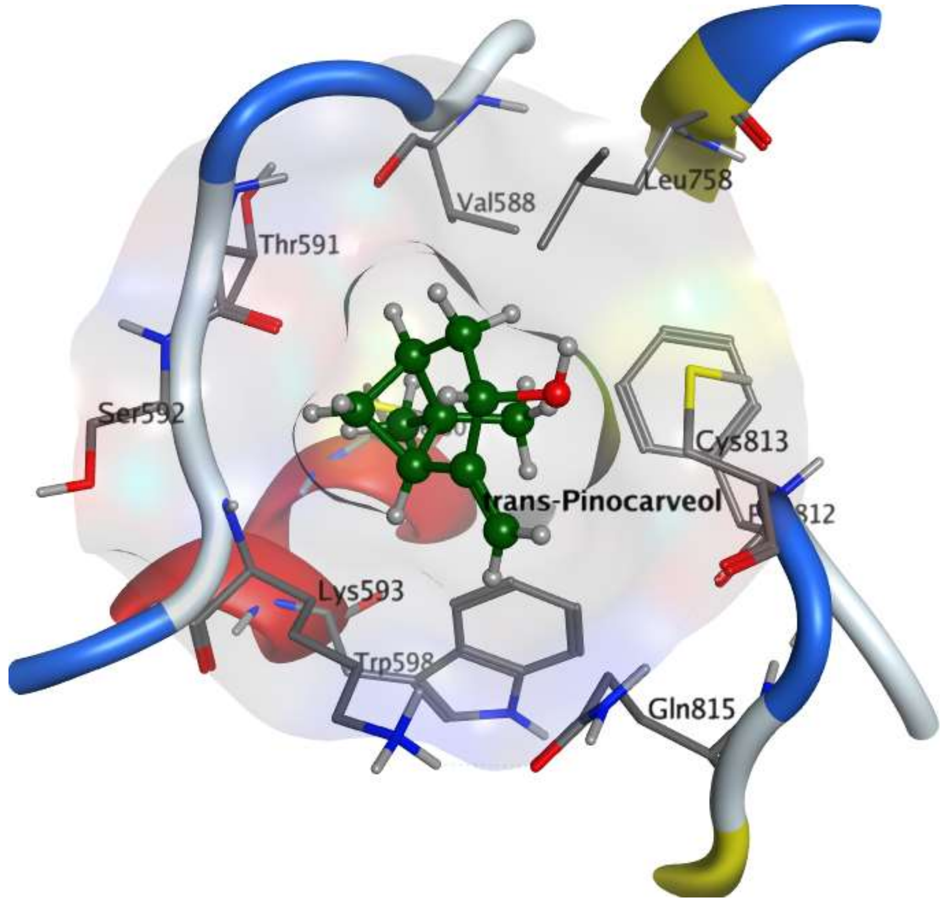	**-4.2639**<br><br>**1.3717**

**Borneol**	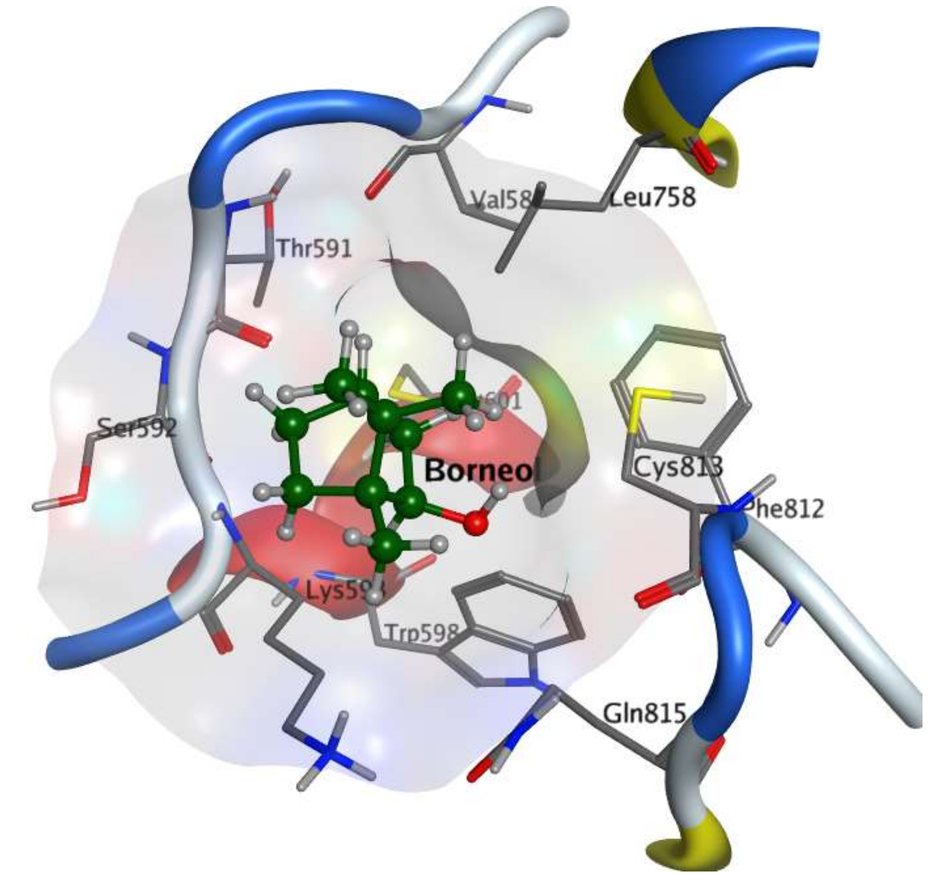	**-4.0815**<br><br>**1.4960**
α-<br>**Phellandren-8-ol**	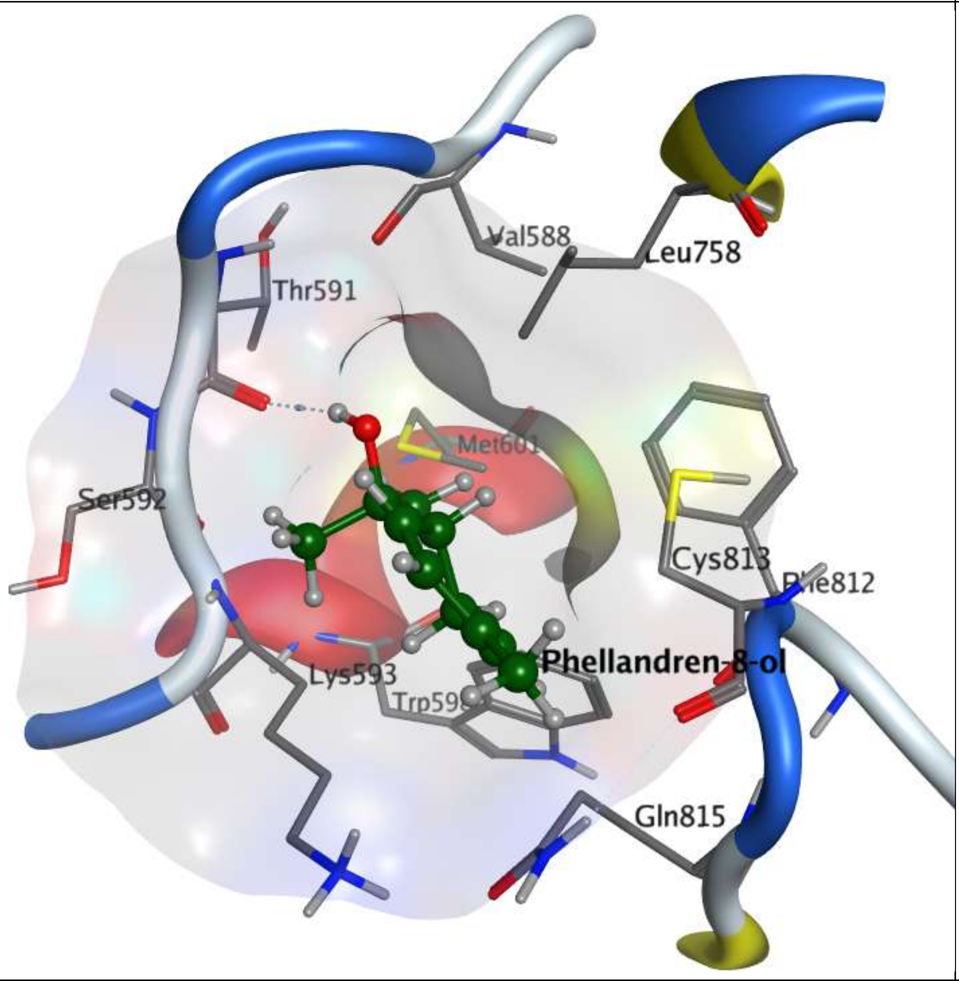	**-4.3839**<br><br>**2.0164**

α-Terpineol	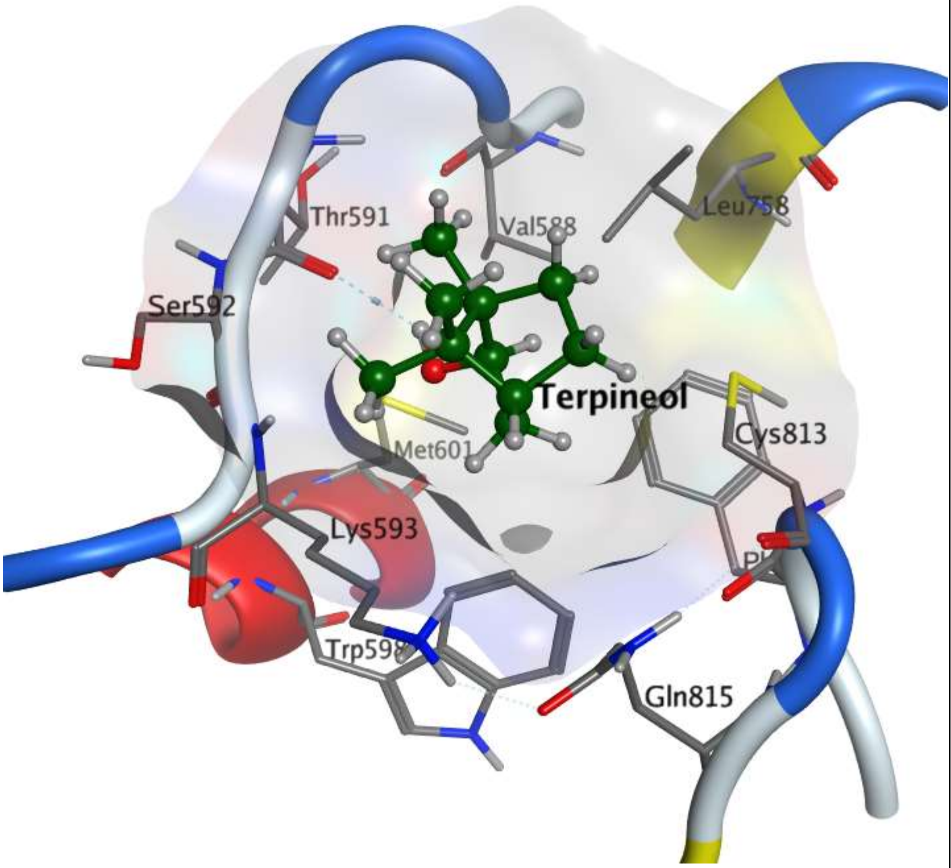	**-4.7213**<br><br>**1.1632**

Table S3. Molecular docking details of *A. robusta* bark EO with RBD.

Component name	Energy score (S) (kcal/mol)	RMSD	Amino acid residues forming	
			**H-bond**	**H-*pi***
**Co-crystallized ligand (NAG)**	-4.5304	1.5097	ASN 343	-
**Tricyclene**	-4.0962	1.4562	-	-
**α-Pinene**	-3.8556	1.1748	-	-
***d*-Camphene**	-4.0584	1.8286	-	-
**Limonene**	-3.9563	1.5847	-	-
***trans*-Pinocarveol**	-4.1561	1.5353	VAL 367	-
**Borneol**	-3.9368	2.1738	ASN 343	-
**α-Phellandren-8-ol**	-4.0385	1.2068	ASN 343	-
**α-Terpineol**	-4.2190	1.9985	ASN 343	-
**2,4-Thujadiene**	-3.9973	1.7303	-	-
**L-β-Pinene**	-3.9127	1.5637	-	-
***m*-Cymene**	-4.1107	1.1504	-	PHE 338
**γ-Terpinene**	-4.2683	1.5342	-	-
**α-Campholenal**	-4.1039	1.3665	-	-
**Camphor**	-4.0149	1.6277	GLY 339	-
**Camphene hydrate**	-3.9441	1.4994	--	-
**Trans-Pinocamphone**	-4.0541	1.8483	-	TRP 436
**Pinocarvone**	-3.9968	1.4778	-	-
**Isoborneol**	-3.9323	1.9775	-	-
***cis*-Verbenol**	-4.0902	1.4764	SER 371	-
**L-terpinen-4-ol**	-4.2719	0.85503		-
**Myrtenal**	-3.9592	1.7403	LEU 368	-
**Verbenone**	-4.1378	1.5315	-	-
***trans*-Carveol**	-4.1642	1.2461	-	-
**(-)-Carvone**	-4.1215	0.9209	-	PHE 342
**Bornyl acetate**	-4.5766	0.9564	-	TRP 436
**α-Terpineol acetate**	-4.7246	0.9855	-	-

Table S4. The target enzymes involved for docking of the major components of *A. robusta* bark EO.

Proteins (PDB)	Resolution	Cognate Ligand	Structure of the cognate ligand	RMSD	Energy score (S) (kcal/mol)
COVID-19 main protease in complex with an inhibitor N3(**6LU7**)	**2.16 Å**	**N3**	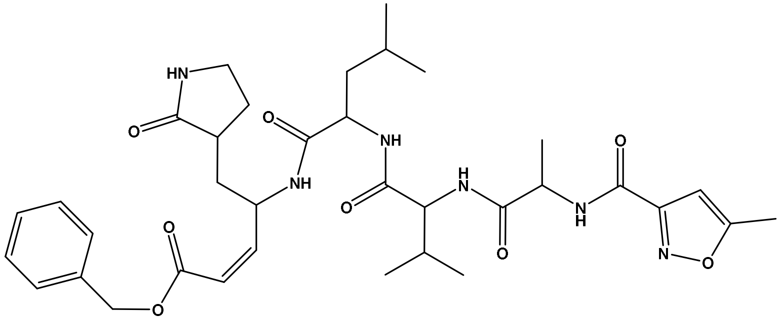	**1.9061**	**-8.4596**
COVID-19 RNA-dependent RNA polymerase bound to suramin: **(7D4F)**	**2.57 Å**	**H3U**	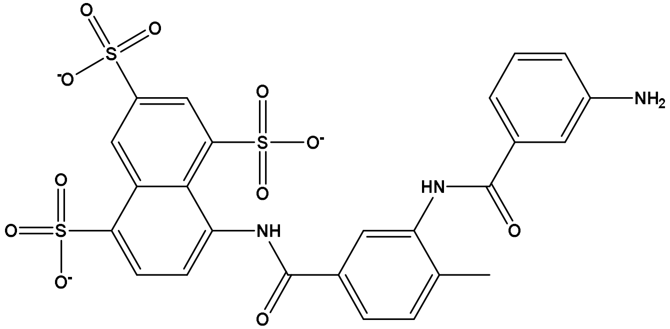	**1.8239**	**-7.7170**
COVID-19 virus spike receptor-binding domain complexed with a neutralizing antibody: **(7BZ5)**	**1.84 Å**	**NAG**	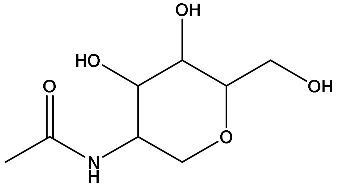	**1.5097**	**-4.5304**
